# Are Asian foods as “fattening” as western-styled fast foods?

**DOI:** 10.1038/s41430-019-0537-3

**Published:** 2019-11-29

**Authors:** Christiani Jeyakumar Henry, Bhupinder Kaur, Rina Yu Chin Quek

**Affiliations:** 1 Clinical Nutrition Research Centre (CNRC), Singapore Institute for Clinical Sciences (SICS), Agency for Science, Technology and Research (A*STAR) Singapore and National University Health System, 30 Medical Drive, Singapore, 117609 Singapore; 2 Department of Biochemistry, Yong Loo Lin School of Medicine, National University of Singapore, S14 Level 5, Science Drive 2, Singapore, 117543 Singapore

**Keywords:** Nutrition, Lifestyle modification

## Abstract

In Asia, the consumption of western-styled fast foods is widely perceived as the cause of the rise in obesity and chronic disease. Twenty-five of the most popular local Asian foods were compared for energy, total fat, saturated fat, sodium, and cholesterol with twenty-nine western-styled fast foods. The comparative analysis showed no significant difference in energy (*p* = 0.150) and total fat (*p* = 0.346) between the two food categories. These findings suggest that many local Asian foods contribute as much energy and total fat in a single meal as western-styled fast foods. Local Asian foods had greater amounts of sodium (*p* < 0.001), saturated fat (*p* = 0.007), and cholesterol (*p* = 0.009) than western-styled fast foods. The persistent presumption that the consumption of western-styled fast foods is the cause of obesity in Asia needs to be challenged. This observation that local Asian foods are as energy dense as western-styled fast foods, will enable us to redress the necessary strategies to address the Asian diet-health debate.

## Introduction

There is widespread presumption that the increase in obesity, cardiovascular disease, and hypertension in Asia is driven by the overconsumption of western-styled fast foods [1]. In parallel, there continues to remain an urban myth that local Asian foods are both healthy and nutritious. We wish to report that both these perceptions are false. This observation is based on a comparative analysis of the energy, total fat, saturated fat, cholesterol, and sodium content of the most commonly consumed foods in Asia (Singapore) with western-styled fast foods.

Singapore is a microcosm of dietary diversity representing Chinese, Indian, and Malay cuisines. These three cuisines encompass the dietary habits of ~4.5 billion people in Asia [2]. Surrounded by a plethora of local food eateries within easy walking distance of most homes and workplaces, Singaporeans eat out regularly [3]. Despite fast food chains being easily accessible in Singapore, an examination of the frequency of fast food consumption shows a counter-intuitive pattern. In a population-based survey, 37% were nonconsumers, 43% were occasional consumers (less than once a week but more than once a month) and 20% consumed fast foods at least once a week [4]. A similar low frequency of western-styled fast food consumption was also reported in Malaysia, Indonesia, and the Philippines [5]. This indicates that the penetration and consumption of western-styled fast foods in this region still remains low. In contrast, the consumption of local foods remains a major source of nutrient intake in Asia [5]. This makes local Asian foods a significant source of energy, sodium, total fat, saturated fat and cholesterol compared with western-styled fast foods. In order to test this hypothesis, we collated a selection of widely consumed local Asian foods and compared their energy, sodium, total fat, saturated fats, and cholesterol values with popular western-styled fast foods (Table 2a, b, Supplementary Files) [6].

## Materials and methods

### Data collection

#### Local Asian foods

Twenty-five of the most commonly consumed local Asian foods from hawker centres and food courts in Singapore were chosen for this analysis. The choice of foods selected was based on interviews/questionnaires on the most widely consumed foods by the three major ethnic groups in Singapore, namely the Chinese, Malays, and Indians [6]. The description of the local Asian foods has been provided in Table 1 (Supplementary file). One of the most commonly consumed rice accompaniments is chicken and this is reflected in the choice of our local Asian foods. In addition, due to portion control, portion sizes are tightly controlled between local vendors. Our previous work also demonstrated that most local foods purchased from various locations had similar energy content [7, 8]. Sodium, total fat, saturated fats, and cholesterol were computed using the “Energy and Nutrient Composition of Food” database by the Health Promotion Board, Singapore [9].

#### Western-styled fast foods

Twenty-nine popular western-styled fast foods from McDonalds’, Pizza Hut, and KFC were chosen and their energy value, total fat, saturated fat, sodium, and cholesterol were computed using food composition tables and their nutrient content obtained from the representative fast food websites.

#### Calculation and statistical analysis

The energy value, total fat, saturated fat, sodium, and cholesterol of local Asian foods and western-styled fast foods were collated based on the serving size of each food (Supplementary Tables 2a, b). Unpaired *t* test (unequal variance) was used to test for the difference in mean between local Asian foods and western-styled fast foods. The alpha (α) level for all statistical analyses in this study was set at 0.05. Values were reported as mean ± standard error of mean. All statistical analyses were performed using Statistical Package for the Social Science (SPSS version 24).

## Results and discussion

**Fig. 1:**
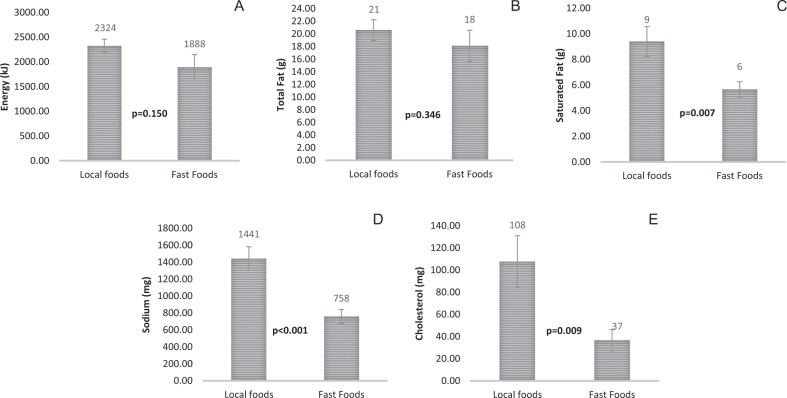
Average (**a**) energy (kJ), (**b**) fat (g), (**c**) saturated fat (g), (**d**) sodium (mg) and (**e**) cholesterol (mg) of 25 local Asian foods and 29 western fast foods (mean ± SEM) (Detailed breakdown of foods analyzed is shown below; nutritional values of individual foods are provided in Supplementary file). Foods analyzed: Local Asian foods. Chinese: Roasted chicken rice, steamed chicken rice, roasted chicken rice (skinless), braised chicken rice, braised chicken rice (skinless), fried kway teow, beef hor fun, fried seafood hor fun, char siew fried rice, char siew rice, Malay: Nasi lemak with chicken wing, nasi lemak with fried egg, mee siam, mee soto, mee goreng, Lontong with sayur lodeh, Indian: Thosia, masala, roti prata (plain), chicken murtabak, chicken briyani, mutton briyani, vegetable briyani, mee goreng (mamak style). Western fast foods. McDonalds extra value meals (Medium fries and Small Coke): Big Mac, Cheeseburger, McChicken, McSpicy, Fillet O Fish, Grilled Chicken McWrap. KFC (Singapore data): 2/3 pcs chicken drumstick + 1 regular whipped potato + 1 regular Coleslaw + 1 regular Pepsi, Shrooms fillet burger/zinger/BBQ Pockett + 1 regular fries + 1 regular Pepsi. Pizza Hut (HPB database): Chicken curry pizza, thin crispy pepperoni pizza, thin crispy supreme pizza, thin crispy cheese pizza, thin crispy super supreme pizza, cheese pan pizza, pepperoni pan pizza, supreme pan pizza, super supreme 9” regular pan pizza, veggie lover’s 10” regular crispy thin pizza, veggie lover’s 12” large pan pizza, veggie lover’s 9” regular pan pizza, shrooms 10” regular crispy thin pizza, chic delite 10” regular crispy thin pizza, hawaiian 12” large pan pizza, hawaiian 9” regular pizza, ocean catch pizza, and meat galore 10” regular crispy thin pizza

The energy value, total fat, saturated fat, sodium, and cholesterol of local Asian foods and western-styled fast foods are displayed in Fig. 1.

There were no significant differences in energy content (*p* = 0.150) and total fat (*p* = 0.346) between local Asian foods and western-styled fast foods. Indeed, several Asian local foods contributed as much energy and total fat in a single meal as western-styled fast foods. Local foods had significantly higher saturated fats (*p* = 0.007), sodium (*p* < 0.001) and cholesterol content (*p* = 0.009) than western-styled fast foods. The higher saturated fat and cholesterol content of Asian foods was predominantly due to animal fats such as lard, fatty meats (pork, beef, and mutton) and skin of poultry (chicken). The higher sodium levels in local Asian foods may be attributed to the use of seasonings such as soya sauce and MSG (monosodium glutamate).

Admittedly, we do not know everything about the relationship between diet and human health. However, in the age of evidence-based science, this communication is intended to provide new insights into the diet-health debate in Asia. Our results highlight the need to reexamine the notion that the consumption of western-styled fast foods alone is the bane of our ill health in Asia. It further demonstrates that Asian foods have unhealthy levels of energy, total fat, saturated fat, sodium and cholesterol. The continuous allegation that consumption of western-styled fast food as the cause of obesity in Asia needs to be reexamined. Asian foods are as high in energy content, saturated fat, sodium and cholesterol as western-styled fast foods. This new observation will facilitate crafting an alternative framework needed to improve the health of the Asian community.

## Supplementary information

Table S1

Table S2a

Table S2b

## References

[1] Guldan GS (2010). Asian children’s obesogenic diets—time to change this part of the energy balance equation?. Res Sports Med.

[2] Liu C, Schänzel H. Introduction to tourism education and Asia. Tourism education and Asia: Springer Nature Singapore Pte Ltd., 2019;p. 3–11.

[3] Story M, Kaphingst KM, Robinson-O’Brien R, Glanz K (2008). Creating healthy food and eating environments: policy and environmental approaches. Annu Rev Public Health.

[4] Whitton C, Ma Y, Bastian AC, Chan MF, Chew L (2014). Fast-food consumers in Singapore: demographic profile, diet quality and weight status. Public Health Nutr.

[5] Naidoo N, van Dam RM, Ng S, Tan CS, Chen S, Lim JY (2017). Determinants of eating at local and western fast-food venues in an urban Asian population: a mixed methods approach. Int J Behav Nutr Phys Act.

[6] Chen L-W, Low YL, Fok D, Han WM, Chong YS, Gluckman P (2014). Dietary changes during pregnancy and the postpartum period in Singaporean Chinese, Malay and Indian women: the GUSTO birth cohort study. Public Health Nutr.

[7] Quek Rina, Goh Hui Jen, Henry CJK (2019). Energy Density of Ethnic Cuisines in Singaporean Hawker Centres: A Comparative Study of Chinese, Malay and Indian Foods. Malaysian Journal of Nutrition.

[8] Lau E, Goh HJ, Quek R, Lim SW, Henry J (2016). Rapid estimation of the energy content of composite foods: the application of the Calorie Answer. Asia Pac J Clin Nutr.

[9] Health Promotion Board. (2003). Food composition guide Singapore.

