# Supplementary material for: Are Asian foods as “fattening” as western-styled fast foods?
Source: Eur J Clin Nutr. 2019 Nov 29;74(2):348–50. doi: 10.1038/s41430-019-0537-3 (PMC7007410; doi:10.1038/s41430-019-0537-3)
Supplement: Supplementary file 1 — Table S1 [file 41430_2019_537_MOESM1_ESM.docx]

**Table S1.** Description of local foods analysed

| *Local Foods* | *Description* |
| --- | --- |
| *Chinese* |  |
| Roasted chicken rice | Roasted chicken with skin, served with flavored rice and chili sauce |
| Steamed chicken rice | Steamed chicken with skin, served with flavored rice and chili sauce |
| Roasted chicken rice (skinless) | Roasted chicken without skin, served with flavored rice and chili |
| Braised chicken rice | Braised chicken with skin, served with flavored rice and chili |
| Braised chicken rice (skinless) | Braised chicken without skin, served with flavored rice and chili |
| *Fried kway teow* | Rice noodles, stir fried in sweet sauce with cockles and Chinese sausage |
| Beef hor fun | Flat rice noodles fried with thinly sliced beef and vegetables served in thick sauce |
| Fried seafood hor fun | Flat rice noodles fried with seafood and vegetables served in thick sauce |
| Char siew fried rice | Rice fried with barbecue pork cubes (char siew) |
| Char siew rice | Sliced barbecue pork served with white rice |
|  |  |
| Malay |  |
| Nasi lemak with chicken wing | Rice dish cooked in coconut milk and pandan leaf served with chicken wing and sambal chili |
| Nasi lemak with fried egg | Rice dish cooked in coconut milk and pandan leaf served with egg and sambal chili |
| Mee Siam | Rice vermicelli, hard-boiled egg, with a sweet and tart gravy |
| *Mee rebus* | Thick yellow egg noodles served in thick spicy gravy together with hard-boiled egg |
| Mee soto | Thick egg noodle soup with Indonesian spicy chicken broth |
| Mee Goreng | Spicy fried yellow egg noodle coated in a thickened sweet and spicy sauce, tossed with fried bean curd cubes, tiger prawns, tomato wedges, leafy vegetable, bean sprout and green onions |
| Lontong with sayur lodeh | Rice cakes cooked with vegetables in a coconut milk-based soup |
|  |  |
| Indian |  |
| Thosai masala | South Indian snack made from fermented pulses |
| *Roti prata* (Plain) | Thick, flat, round dough made with wheat flour, sugar, salt and water, fried with ghee |
| *Chicken murtabak* | Pan-fried folded flatbread stuffed with chicken and vegetables |
| Chicken biryani | Rice cooked with ghee and spices, served with spicy chicken |
| Mutton briyani | Basmati rice flavored with spices and served with mutton |
| Vegetable briyani | Basmati rice flavored with spices and served with vegetables |
| Mee goreng | Spicy stir fried egg noodle |
