# Supplementary material for: Are Asian foods as “fattening” as western-styled fast foods?
Source: Eur J Clin Nutr. 2019 Nov 29;74(2):348–50. doi: 10.1038/s41430-019-0537-3 (PMC7007410; doi:10.1038/s41430-019-0537-3)
Supplement: Supplementary file 2 — Table S2a [file 41430_2019_537_MOESM2_ESM.docx]

Table S2a: Detailed nutritional composition of local Asian foods analysed

| **Ethnic Group** | **Food Item** | **Serving Size (g)** | **Energy (kJ)** | **Total Fat (g)** | **Saturated Fat (g)** | **Carbohydrate (g)** | **Cholesterol (mg)** | **Sodium (mg)** |
| --- | --- | --- | --- | --- | --- | --- | --- | --- |
| Chinese | Chicken rice, with roasted chicken | 330 | 2193 | 19.75 | 7.55 | 64.47 | 40.66 | 1112.23 |
|  | Chicken rice, with steamed chicken | 330 | 2331 | 13.86 | 4.95 | 80.19 | 36.63 | 697.95 |
|  | Chicken rice, with skin removed | 365 | 2654 | 21.75 | 8.51 | 84.10 | 66.03 | 1035.54 |
|  | Braised chicken rice | 309 | 2294 | 23.88 | 7.67 | 59.08 | 33.62 | 835.67 |
|  | Braised chicken rice, skinless chicken | 291 | 2103 | 14.78 | 4.89 | 68.91 | 26.48 | 1009.19 |
|  | Char Kway Teow | 384 | 3114 | 38.40 | 29.18 | 76.03 | 234.24 | 1459.20 |
|  | Beef hor fun | 481 | 2915 | 26.46 | 11.54 | 94.76 | 38.48 | 1717.17 |
|  | Fried seafood hor fun | 693 | 3128 | 27.03 | 11.16 | 90.78 | 76.23 | 1884.96 |
|  | Ipoh hor fun | 449 | 1896 | 13.92 | 4.49 | 66.00 | 71.84 | 1638.85 |
|  | Char siew fried rice | 418 | 2125 | 20.06 | 8.36 | 66.04 | 443.08 | 1621.84 |
|  | Char siew rice | 327 | 2529 | 16.35 | 11.90 | 91.23 | 55.59 | 879.63 |
| Malay | Nasi Lemak with chicken wing | 306 | 2745 | 24.94 | 10.65 | 81.21 | 117.44 | 877.09 |
|  | Nasi Lemak with fried egg only | 210 | 2063 | 13.65 | 7.56 | 79.99 | 76.23 | 837.90 |
|  | Mee Siam | 624 | 2347 | 18.47 | 6.68 | 79.87 | NA | 2808.00 |
|  | Mee Rebus | 571 | 2338 | 18.16 | 3.60 | 78.00 | 146.40 | 2268.87 |
|  | Mee Soto | 541 | 1809 | 12.98 | 5.41 | 60.05 | 37.87 | 2677.95 |
|  | Mee Goreng | 390 | 2087 | 21.37 | 9.40 | 59.67 | NA | 2301.00 |
|  | Lotong with Sayur Lodeh | 775 | 2149 | 26.00 | 14.10 | 41.40 | NA | 1242.50 |
| Indian | Thosai masala | 225 | 1514 | 13.28 | 5.76 | 52.43 | NA | 778.50 |
|  | Roti Prata (plain) | 60 | 680 | 7.14 | 3.38 | 19.50 | NA | 260.4 |
|  | Chicken murtabak | 500 | 3198 | 32.00 | 14.00 | 83.50 | 175.00 | 2470.00 |
|  | Chicken briyani | 400 | 3004 | 28.50 | 12.16 | 83.60 | 129.20 | 1356.60 |
|  | Mutton briyani | 505 | 3143 | 24.75 | 12.12 | 96.46 | 95.95 | 1858.40 |
|  | Vegetable briyani | 239 | 1386 | 13.50 | 8.50 | 45.72 | 54.49 | 606.48 |
|  | Mee Goreng (Mamak style) | 170 | 1177 | 9.01 | 3.67 | 40.63 | 28.73 | 1111.80 |
