# Supplementary material for: Are Asian foods as “fattening” as western-styled fast foods?
Source: Eur J Clin Nutr. 2019 Nov 29;74(2):348–50. doi: 10.1038/s41430-019-0537-3 (PMC7007410; doi:10.1038/s41430-019-0537-3)
Supplement: Supplementary file 3 — Table S2b [file 41430_2019_537_MOESM3_ESM.docx]

Table S2b: Detailed nutritional composition of Western styled fast foods analysed

| **Restaurants** | **Food Item** | **Servings (g)** | **Energy (kJ)** | **Total Fat (g)** | **Saturated Fat (g)** | **Cholesterol (mg)** | **Carbohydrates (g)** | **Sodium (mg)** |
| --- | --- | --- | --- | --- | --- | --- | --- | --- |
| **McDonalds** | Big Mac Extra Value Meal (medium fries & small coke) | NA | 4268 | 43 | 12.9 | 178 | 127 | 1220 |
|  | Cheeseburger Extra Value Meal (medium fries & small coke) | NA | 3340 | 29 | 8.6 | 141 | 116 | 960 |
|  | McChicken Extra Value Meal (medium fries & small coke) | NA | 3695 | 34 | 5.7 | 41 | 127 | 1010 |
|  | McSpicy Extra Value Meal (medium fries & small coke) | NA | 4268 | 42 | 7.6 | 75 | 134 | 1530 |
|  | Fillet O Fish Extra Value Meal (medium fries & small coke) | NA | 3474 | 30 | 6 | 90 | 122 | 820 |
|  | Grilled Chicken McWrap Extra Value Meal (medium fries & small coke) | NA | 3497 | 33 | 6.7 | 81.9 | 110.4 | 1139 |
| **KFC (Singapore data)** | 2 pcs Chicken Meal (drumsticks) + 1 regular whipped potato + 1 regular coleslaw + 1 regular Pepsi | 591.0 | 2157 | 20 | 6 | NA | 65 | 686.0 |
|  | 3 pcs Chicken Meal (drumsticks) + 1 regular whipped potato + 1 regular coleslaw + 1 regular Pepsi | 677.0 | 3043 | 33 | 11 | NA | 73 | 998.0 |
|  | Shrooms Fillet Burger + 1 regular fries + 1 regular Pepsi | 621.0 | 3273 | 23 | 8 | NA | 119 | 1899 |
|  | Zinger+ 1 regular fries + 1 regular pepsi | 640.0 | 4305 | 45 | 14 | NA | 124 | 1772 |
|  | Bandito Pockett + 1 regular fries + 1 regular pepsi | 430.0 | 4259 | 36 | 11 | NA | 49 | 1363 |
| **Pizza Hut (HPB database)** | Chicken curry pizza | 1 slice (80g) | 682 | 7.52 | 3.32 | 12.56 | 13.44 | 426.4 |
|  | Pizza, thin n crispy, pepperoni | 1 slice (75g) | 1044 | 10.2 | 4.57 | 22.5 | 24.98 | 693 |
|  | Pizza, thin n crispy, supreme | 1 slice (100g) | 1078 | 11.5 | 5.2 | 21 | 25 | 740 |
|  | Pizza, thin n crispy, cheese | 1 slice (75g) | 890 | 9.15 | 4.57 | 20.25 | 21.3 | 537 |
|  | Pizza, thin n crispy, super supreme | 1 slice (100g) | 1028 | 11.4 | 4.4 | 26 | 22.8 | 711 |
|  | Pizza, pan, cheese | 100g | 932 | 5.1 | 2.44 | 15 | 32.54 | 533 |
|  | Pizza, pan, pepperoni | 1 slice (95g) | 1136 | 11.59 | 4.37 | 19 | 30.02 | 620.35 |
|  | Pizza, pan, supreme | 130 | 1243 | 14.34 | 5.73 | NA | 26.78 | 730.29 |
|  | Super Supreme 9" Regular Pan Pizza | 78 | 815 | 7.8 | 3.4 | 9.5 | 21.1 | 358.8 |
|  | Veggie Lover's 10" regular crispy thin pizza | 63 | 585 | 4.7 | 3.1 | 4.41 | 18.3 | 270.27 |
|  | Veggie Lover's 12" large pan pizza | 85 | 796 | 6.3 | 3.1 | 7.5 | 25.1 | 353.6 |
|  | Veggie Lover's 9" regular pan pizza | 78 | 701 | 5.5 | 2.4 | 5.46 | 22 | 283.14 |
|  | Shrooms 10" regular crispy thin pizza | 64 | 618 | 5.6 | 3.1 | 6.4 | 16.8 | 353.28 |
|  | Chic Delite 10" Regular Crispy Thin Pizza | 61 | 586 | 4.1 | 2.8 g | 10.98 | 14.51 | 323.91 |
|  | Hawaiian 12" large pan pizza | 82g | 833 | 7.71 | 4.1 | 12.3 | 21.6 | 402.62 |
|  | Hawaiian 9" large pan pizza | 74g | 739 | 6.2 | 2.8 | 8.88 | 21.6 | 330.04 |
|  | Ocean Catch pizza | 1 slice (80g) | 716 | 7.12 | 2.96 | 19.04 | 16.24 | 452 |
|  | Meat Galore 10" Regular Crispy Thin Pizza | 61 | 737 | 8.3 | 4.3 | 13.42 | 13.7 | 462.99 |
